# From Genes to Health – Challenges and Opportunities

**DOI:** 10.3389/fped.2014.00012

**Published:** 2014-03-03

**Authors:** Muhammad Ramzan Manwar Hussain, Asifullah Khan, Hussein Sheikh Ali Mohamoud

**Affiliations:** ^1^Princess Al-Jawhara Al-Brahim Center of Excellence in Research of Hereditary Diseases (PACER-HD), Department of Genetic Medicine, King Abdulaziz University, Jeddah, Saudi Arabia; ^2^Department of Biochemistry, Abdul Wali Khan University, Mardan, Pakistan

**Keywords:** genomics, Mendelian puzzles, pediatric cancer, next-generation sequencing, challenges

## Abstract

In genome science, the advancement in high-throughput sequencing technologies and bioinformatics analysis is facilitating the better understanding of Mendelian and complex trait inheritance. Charting the genetic basis of complex diseases – including pediatric cancer, and interpreting huge amount of next-generation sequencing data are among the major technical challenges to be overcome in order to understand the molecular basis of various diseases and genetic disorders. In this review, we provide insights into some major challenges currently hindering a better understanding of Mendelian and complex trait inheritance, and thus impeding medical benefits to patients.

## Genomics and Mendelian Puzzles

Single-gene mutations in the form of rare clinical phenotypes and Mendelian disorders are being identified in every age and sex group of the human population (1). After characterizing genes and mutations for over 2500 Mendelian disorders in the human genome project (HGP) (2), tremendous progress has been made in developing fast strategies for entire human exome sequencing (3). However, in order to discover the full spectrum of Mendelian phenotypic variations, it is necessary to inspect gene-regulatory sequences more closely. Mutations in either of two different but adjacent genes with cis-regulatory module (i.e., sharing a common regulatory region) in Joubert syndrome provide a good example of how human genetics is expanding from the single-gene concept to a more genomic outlook (4). In the human genome sequence, the widespread conservation of non-coding DNA carries a variety of regulatory elements that either enhance, suppress, or insulate the transcription of genes (5).

In Mendelian genetics, two puzzles have been recognized as a result of mutation analyses of single-gene defects. The first mystery is that not all individuals with a particular disorder have recognizable coding mutations; the second is that not all individuals with the same disease-causing mutation, even within a family, suffer from the disease, and some of them may be completely unaffected (4). The second puzzle is considered as being the more challenging, due to variation in disease penetrance and expressivity. In order to understand disease-phenotypes, scientists are trying to relate phenotypic variations, observed as a result of penetrance and expressivity, with molecular level changes (6–8). For example, why is one member of a family with mutation in *Rb1* gene affected with retinoblastoma (OMIM: 180200) and not the other – although both carry the same mutation (9, 10). Similarly, why does the disease severity for neurofibromatosis (OMIM: 162200) differ significantly between two members of a family harboring the same mutation in the neurofibromin (*NF1*) gene (11). After studying this persistent challenge, scientists have concluded that various contributory factors, including modifier genes (12), environmental factors (4), allelic variations, and complex genetic and environmental interactions, all play a role in regulating variable disease-phenotypes in humans. However, a full and accurate understanding of these factors in order to resolve such Mendelian puzzles is still a work in progress.

In addition to monogenic disorders (which does not mean monocausal), understanding the genetic basis of complex modes of inheritance relating to diseases of digenic, trigenic, and even more complex traits, is another challenge in genome science. In analyzing complex modes of inheritance, genome-wide association study (GWAS) strategies have not lived up to earlier expectations for the discovery of complex traits, due to the erroneous assumption that common disorders are regulated by common genetic risk factors (13). The systematic elucidation of monogenic disorders by means of genomic research is an important tool for characterizing the function of genes and of course the whole genetic architecture (13, 14). The scientific community believes that whole-genome sequencing may resolve the dilemma of more complex inheritance in the near future (3).

## Clinical Benefits and Genetic Diversity – A Major Challenge

Population genetic studies are important for understanding the distribution of genetic variability among populations, inferring their demographic histories and their adaptation to natural selection and genes flow, associated with human diseases and health (15). Moreover, in medical research, genetic diversity has become both a challenge and an opportunity for understanding specific genetic factors, facilitating improvements in diagnosis, risk factor identification, differential treatment, and eventually, in more effective cures and the prevention of human disease (16). The achievement of entire human genome sequence information during the HGP and its combination with the international HapMap project has characterized approximately 10 million common variants in different world populations (17). To develop a greater understanding of low-frequency and rare human genomic variations, a project dealing with the sequencing of 1000+ individuals from different population has already been conducted (2). The knowledge of the involvement of genetic and epigenetic factors in disease and the interpretation of associated cellular and biological processes, are the principal tools to translate genomic discoveries into novel therapeutic approaches in medical practice. However, there are still considerable barriers to overcome, and the anticipated improvements in the effectiveness of health care may not be achievable until sometime in the next decade (Najib Al-Khaja, Centre for Arab Genomic Studies) (13).

Today, clinicians are faced with the difficulty of choosing between disease-targeted sequencing tests and high-throughput sequencing (genome and exome sequencing) approaches (18). Within the scientific community, people still need a deeper understanding of human genome biology and bioinformatics tools to analyze huge datasets to interpret the genetic variants in order to approach differential diseases diagnosis and therapies (19, 20). However, in the future, the cost-effective personal genome sequencing of an individual, and the interpretation of sequence variation into biologically meaningful conclusions, is likely to become a standard component of health care.

## Pediatric Cancer Genome Project

In developed countries, cytotoxic chemotherapy and radiotherapies have raised the overall success rate in the treatment of pediatric cancer to 80%. However, a great reduction in life quality due to major side-effects of therapies is limiting the success of therapy (21–23). During early age organ development and maturation, the spectrum of mutations causing malignant transformations results in notable differences in the spectrum of cancers between children and adults (24, 25). Understanding the genetic abnormalities underlying adult and pediatric cancers is an essential step in developing novel drug therapies for cancers, and especially so for pediatric cancers. In 2010, an effort was compiled in the form of the Pediatric Cancer Genome Project (PCGP), established by St. Jude Children’s Research Hospital and the Genome Institute at Washington University, and with the aim of exploring both inter- and intra-chromosomal rearrangements, and the mechanisms of mutagenesis. The PCGP should not only provide a unique understanding of modified signaling pathways in cancer but also indicate improved ways of achieving vital therapeutic targets, especially for children (26).

The presence of structural variations in repetitive DNA sequences, heterogeneity within tumor samples, and mutations affecting the regulatory regions of genes are the main foci of PCGP’s aim of better understanding the complications of genome data interpretation. The human reference genome still carries gaps and there is an incomplete picture of the full set of genome variations in human population groups (26). Hence, in a wide range of projects, paired-end sequencing technology is still struggling to create a full picture of structural variations in repetitive DNA sequences (26, 27).

The striking degree of diversity in human tumor samples has resulted in the initiation of a number of different cancer projects aimed at developing the accurate estimation of cancer cells intermixing with normal support and immune cells (26). Finally, mutation-profiling studies (frequency and functional consequences using large numbers of samples) in the large conserved DNA regions need precise analytical methods to investigate mRNAs, non-coding RNAs, and epigenetic data on CpG methylation.

## Challenges to Next-Generation Sequencing Analysis

Over the last decade, numerous advances in the fields of structural and functional genomics have taken place (20). High-throughput next-generation technologies are being widely used to explore such fields, aiming to complete the diagnostic odyssey in search of cost-effective techniques (18). The scale and efficiency of sequencing is being harnessed by researchers to address specific clinical problems, and to more fully understand the complexities of human biology and the place of epigenetic mechanisms (16).

After microarray technology, next-generation sequencing (NGS) technology has made the problem of data analysis more challenging by generating substantial data in the form of whole-genome sequencing, exome sequencing, transcriptome analysis, expression profile chromatin immunoprecipitation-based (ChIP) sequencing, and methylome (epigenetics) (18). In NGS, genome level alignment and short reads assembling of huge data volumes carry technical challenges to get accurate identification of variants base calling in the form of SNPs, SNVs, and indels, especially at the repetitive loci of human genome (28). IGV and SAM tools are being used to resolve the erroneous alignment problem of multi-reads, manually. However, this is not usually a feasible strategy for very large NGS datasets. SNP calling using GATK, MAQ, SAMtools, SOAPsnp, or VarScan programs is the next approach in computational pipeline after reads mapping. The accurate *de novo* assembly of short reads, at repetitive loci, as delivered by most NGS technologies, is challenging. In addition to creating gaps, complex and misassembled rearrangements could be resulting from the erroneous collapse of repeats on one another. Consequently, the accurate and scalable assessment of structural variations solely by NGS platform is still problematic. (20). Despite these challenges, two class of *de novo* assemblers, i.e., overlap-based assemblers and de Bruijn graph assemblers, have been developed to tackle this problem (28, 29). After collection and data interpretation, the next challenge is in accessing and sharing large scale data to the researchers for further comparative genome analysis. However, in disease genomics, the development of cutting-edge algorithms for quick data analysis of NGS datasets using table computers may be helpful to bring potential medical benefits to patients.

## Conclusion

Recent progress in acquiring complete genome sequence information is leading toward a personalized medicine tradition. Although whole genome and exome sequencing strategies are contributing to anticipate clinically significant variants, these are still in infancy. The accurate understanding of factors influencing rare Mendelian and common genetic traits, and the precise handling of high-throughput next-generation data, are the most significant technical challenges still impeding the medical benefits to patients. However, the cost-effective application of NGS technologies – quick, secure, accurate analysis of large scale NGS data – for identification of common and rare genetic variants across the human populations and diseased individuals together with advancement in the fields of proteomics and metabolomics will provide a way of knowledge transfer from scientific literature toward clinical practices.

## Conflict of Interest Statement

The authors declare that the research was conducted in the absence of any commercial or financial relationships that could be construed as a potential conflict of interest.
